# Oncogenic senescence: a multi-functional perspective

**DOI:** 10.18632/oncotarget.15742

**Published:** 2017-02-25

**Authors:** Darren J. Baker, Fatouma Alimirah, Jan M. van Deursen, Judith Campisi, Jeffrey Hildesheim

**Affiliations:** ^1^ Department of Pediatric and Adolescent Medicine, Mayo Clinic, Rochester, Minnesota, USA; ^2^ Department of Biochemistry and Molecular Biology, Mayo Clinic, Rochester, Minnesota, USA; ^3^ Buck Institute for Research on Aging, Novato, California, USA; ^4^ Division of Cancer Biology, National Cancer Institute, National Institutes of Health, Bethesda, Maryland, USA

**Keywords:** cellular senescence, tumorigenesis, microenvironment

## Abstract

Cellular senescence is defined as an irreversible growth arrest with the acquisition of a distinctive secretome. The growth arrest is a potent anticancer mechanism whereas the secretome facilitates wound healing, tissue repair, and development. The senescence response has also become increasingly recognized as an important contributor to aging and age-related diseases, including cancer. Although oncogenic mutations are capable of inducing a beneficial senescence response that prevents the growth of premalignant cells and promotes cancer immune-surveillance, the secretome of senescent cells also includes factors with pro-tumorigenic properties. On June 23^rd^ and 24^th^, 2016, the Division of Cancer Biology of the National Cancer Institute sponsored a workshop to discuss the complex role of cellular senescence in tumorigenesis with the goal to define the major challenges and opportunities within this important field of cancer research. Additionally, it was noted how the development of novel tools and technologies are required to accelerate research into a mechanistic understanding of senescent cells in carcinogenesis in order to overcome the current limitations in this exciting, yet ill-defined area.

## INTRODUCTION

Cellular senescence is traditionally viewed as an autonomous, tumor-suppressive mechanism that irreversibly blocks cellular proliferation in response to a variety of stresses such as DNA damage, telomere attrition or oncogene activation [1, 2]. While significant attention is given to further elucidate the biological benefits of senescence, the understanding of its detrimental effects is also evolving, along with non-stress related functions. Senescent cells have been shown to promote neoplastic transformation and their elimination is found to delay tumor formation [1, 3]. Furthermore, senescent cells are genomically unstable and cancer therapy-induced senescence is associated with invasive and metastatic phenotypes in certain cancer types [1, 4]. Developmental forms of cellular senescence have been identified, which do not seem to be stress-induced and function in normal tissue growth and remodeling programs [5, 6].

Some of the key challenges to the narrowly structured, cell autonomous model of cellular senescence is that its induction in response to stress or developmental cues is not mediated by senescence-specific events, but rather represents a collection of cellular functions that may or may not occur in concert [1, 2]. Senescence, for instance, involves cell cycle arrest, DNA damage responses, epigenetic and nuclear envelope changes, along with autophagy and metabolic reprogramming that are often associated with other cellular events [1, 2]. Nevertheless, these cellular programs are either directly or indirectly involved in driving a complex and highly plastic senescence-associated secretory phenotype (SASP) response [1, 4]. The SASP is comprised of numerous chemokines, growth factors, cytokines, matrix metalloproteinases and small molecular weight metabolites [4]. From an aging perspective, senescent cells are known to accumulate over time, and increased numbers of senesced cells (and their SASP) are suspected to contribute to late-life deterioration of tissues and organs and drive age-related diseases, including cancer [1, 2]. It is currently unclear how dynamic the SASP is over time/age and how it may differ in response to different oncogenic or developmental signals. Furthermore, the composition of the SASP likely varies depending on the cell type undergoing senescence in different tissues. It is also unclear whether the SASP has beneficial and/or deleterious contributions to tissue homeostasis and/or cancer progression, as it may be extremely context dependent [7]. Paradoxically, senescence is seemingly preferred to apoptosis in response to oncogenic stimuli, and the genetic background of cells subjected to stress conditions may preferentially trigger senescent and/or apoptotic pathways, but the evolutionary advantage for this predilection remains poorly understood [8].

In light of its multi-dimensional functions, alternative regulatory pathways and context-specific effects, it is becoming increasingly clear that there may be multiple classes of senescent cells, ranging from acute (programmed) senescent cells to chronic (spontaneous age-related) and disease-related senescent cells, along with various forms of tumor-related senescent cells such as senescent cancer cells, senescent stromal cells, therapy-induced senescent cells, and bystander senescent cells [9]. Each of these may consist of several senescent cell subclasses that may be in different stages of induction (early versus late/deep senescence), illustrating the immense complexity of the problem of establishing how senescence contributes to cancer or other age-related disorders.

As the field of senescence biology matures, so is the awareness of its inherent complexities and potentially harmful effects. From a cancer biology standpoint, important questions about the senescence-associated benefits and detriments have emerged – including questions about its significance (and involvement) in tumor initiation, its contribution to tumor heterogeneity and resistance to therapy, and the extent to which senescent cells are necessary for normal organ function/homeostasis or whether they persist and remain irreversibly cell cycle arrested in aging host tissues. These salient questions are accompanied by the realization that critical tools are missing, for instance, to fate map these cells *in vivo*, or to catalog their unique SASP over time or eliminate them in a spatiotemporal manner.

To assess the impact of senescent cells on cancer, the Division of Cancer Biology (DCB) at the National Cancer Institute (NCI) organized a workshop in explore the role of the senescent cells to tumor initiation and promotion. A panel of experts, chaired by Judy Campisi (Buck Institute) and Jan van Deursen (Mayo Clinic), summarized the current state of the field (including its limitations) and highlighted promising research directions and opportunities that could enhance our understanding of how senescent cells both suppress and contribute to cancer. The meeting summary highlights research priorities and aims to stimulate basic and translational research to generate a better understanding of the molecular mechanisms regulating oncogenic senescence, with the ultimate goal to leverage insights gained for improved cancer therapies.

## SENESCENCE AS A MULTIFACETED PROGRAM

Cellular senescence likely evolved as a tumor suppressive program by which damaged cells irreversibly arrest proliferation to halt progression to malignancy. Now, however, senescence is emerging as a more complex physiological phenomenon observed at all stages of life and with far reaching consequences. Senescent cells have now been observed in several diverse biological processes, including embryogenesis [5, 6], tissue remodeling, wound healing [7, 10] and chromatin reorganization [11]. In this session, the detrimental and beneficial roles of senescence and the SASP were discussed.

**Bill Keyes** (Center for Genomic Regulation; Barcelona, Spain) discussed the complex effects of the SASP on cellular plasticity, tissue regeneration, and tumor growth. Recent developments in the Keyes Laboratory show that induction of oncogene-induced senescence paradoxically coincides with increased expression of tissue specific stem cell markers in primary mouse keratinocytes. Extending this to an *in vivo* model of senescence, the DMBA/TPA-induced skin carcinogenesis model further supported that senescent cells express increased stem cell markers, including CD34, a marker of hair follicle bulge stem cells. These cells not only are required for hair follicle regeneration, but also represent the cell of origin in papilloma and squamous cell carcinomas (SCCs). To further explore the link between senescence and stemness, Ras-induced keratinocytes undergoing senescence were transplanted into full thickness wounds in nude mice. Remarkably, this transplant model also resulted in papilloma formation, which were identical to DMBA/TPA induced lesions, including an aberrant layer of CD34^+^ cells. However, tracing of the transplanted senescing cells revealed that not all of the papilloma arose from the grafted cells, and included regions recruited from the endogenous keratinocytes. This suggests that the SASP may play a role in directing papilloma development.

In support of this observation, they next identified that transient (2 days) exposure of normal mouse primary epidermal keratinocytes to conditioned media (CM) from oncogene-induced senescent cells also results in an increase in CD34^+^ cells. Accordingly, the transiently conditioned keratinocytes were capable of re-epithelializing full-thickness wounds upon *in vivo* transplantation onto the dorsal surface of athymic nude mice and of regenerating fully functional hair follicles. This suggests that components of the SASP can induce stem cell functionality and regenerative capacity. Curiously however, these cells also expressed increased senescence-associated β-galactosidase (SAβG), but not p16^Ink4a^. In stark contrast, chronic CM/SASP exposure (7 days), while increasing stem cell marker gene expression further, now caused a cell-intrinsic paracrine arrest of the cultured normal primary cells. This suggests that the SASP can induce beneficial plasticity, but when prolonged, one which is blocked by a cell-intrinsic senescence arrest.

Collectively these results suggest that non-cell autonomous effects of the SASP can induce cell plasticity by initially driving keratinocyte dedifferentiation, but when prolonged, this is sensed as aberrant or tumor-promoting, and is counteracted by a cell-intrinsic senescence arrest. This SASP-induced plasticity appears to be essential during tissue regeneration/wound healing but also repurposed (and usurped) during papillomagenesis. The extent to which regeneration- and tumorigenesis-based senescence are similar and/or distinct remains to be determined.

The discussion on SASP-induced cellular plasticity was continued by **Manuel Serrano** (Spanish National Cancer Center (CNIO); Madrid, Spain). He first argued that cell culture and *in vivo* cellular processes (including senescence) likely have more similarities than differences – and that true tissue culture artifacts are unlikely to be as pervasive as many believe them to be. He then introduced his work on how senescence contributes to *in vivo* cell plasticity and repair programs in an evolutionarily advantageous manner by inducing a pro-inflammatory response, unlike apoptosis. Cellular reprogramming has been well established and characterized *in vitro* using the Yamanaka transcription factors, which includes Oct4, Sox2, Klf4, and c-Myc [12]. To test the consequences of cellular reprogramming *in vivo*, the Serrano laboratory developed a tetracycline-inducible mouse (i4F) to ubiquitously express the four Yamanaka transcription factors (13). Treatment of adult mice with doxocycline for 1-3 weeks results in widespread dysplasia, along with loss of cell identity (in a process defined as “de-differentiation”), and detection of circulating induced pluripotent stem cells (iPSCs) in the blood. Furthermore, elevated levels of Nanog (a marker of undifferentiated stem cells) were noted, along with SAβG activity and increased p21^Cip1^ in the liver, kidney, stomach, pancreas and intestine – albeit Nanog and senescence markers are not expressed in the same cells. Curiously, this i4F-mediated effect is not observed in the lung, unless first exposed to the DNA damaging agent, bleomycin. After ∼4 weeks of constant doxycycline exposure, the mice die of intestinal hyperplasia. Nevertheless, if doxycycline is removed, full recovery is achieved after two weeks. However, ∼2 months out from doxycycline cessation, a few reprogrammed cells continue to grow and ultimately develop into teratomas and other types of cancer. [13]. It has been shown that p53 and p16^Ink4a^ are barriers to cellular reprogramming *in vitro*. To determine the relationship between cellular reprogramming and senescence *in vivo*, i4F mice were created that lack either one of these important regulators. Deletion of p53 promotes higher rates of reprogramming, teratoma formation, and paradoxically senescence. Interestingly, reprogrammed cells (i.e., positive for Nanog) are located in close proximity to senescent cells (ie., positive for SAβG or p21^Cip1^), but there is no indication that these cells exhibit any senescent cell characteristics. Loss of p16^Ink4a^/p19^Arf^ on the other hand limits reprogramming and senescence in i4F mice, which leads to fewer teratomas. These results suggest that p16^Ink4a^ is required for *in vivo* reprogramming-induced senescence. Furthermore, inflammation and the SASP are essential components of this process, as treatment with IKK and PIM-1 inhibitors or αIL6 antibodies reduce the reprogramming efficiency, as does treatment with ABT-263 (Navitoclax, a first generation senolytic). Conversely, treatment with palbociclib, a selective CDK4/6 inhibitor that promotes senescence, has the opposite effect. As the i4F mice do not express the reprogramming factors in the lungs, the animals were first treated with bleomycin to induce senescence, followed by the administration of doxycycline. Only bleomycin-treated lungs (which harbor senescent cells) undergo reprogramming. To substantiate this finding, they also observed that older mice reprogram more efficiently than younger mice because old mice harbor a senescence-rich microenvironment, which highlights the importance of senescence in this process. In keeping with his introduction, Dr. Serrano concluded by pointing out that leukemia inhibitory factor (LIF) is a critical (but often overlooked) factor for cellular reprogramming. While it is not one of the Yamanaka factors, LIF is related to IL6, suggesting that IL6 may be required for efficient reprogramming in both cultured cells and *in vivo* platforms.

**Ana Banito** (Memorial Sloan Kettering Cancer Center; New York, USA) concluded this session by focusing on how senescent cells remodel the enhancer landscape to control the SASP [11]. Using oncogene-induced senescent IMR90 (human fetal lung) fibroblasts, they found that, unlike quiescence, Ras^G12D^-induced senescence results in appreciable changes in the typical enhancer and super enhancer profiles as determined through H3K27Ac ChIP-seq analysis. Of the >32,000 H3K27Ac-marked enhancers identified, 1,255 are super enhancers. Of these, a subset of 191 are inactivated, including many in proximity to proliferation genes such as E2F target genes. Furthermore, they found that 198 super enhancers are activated in senescence, many of which are near SASP genes, including IL8 and IL1α. Moreover, the chromatin reader BRD4 was identified as a regulator of the SASP and its downstream paracrine signaling. In senescent cells, BRD4 is highly enriched at super enhancers near SASP genes, including IL1α, IL1β, IL8, MMP-1 and MMP-10, suggesting that BRD4 is an important regulator of the inflammatory features of the SASP. Furthermore, *in vivo* BRD4 ablation (via shRNA) or inhibition (via BET inhibitors) markedly decreases the SASP and inhibits the cytotoxicity of natural killer (NK) cells without affecting other features of the senescent phenotype such as cell growth arrest. In line with its role as a regulator of the SASP, BRD4 was also found to be essential in maintaining tumor suppressive immune surveillance [11]. This study underscores the importance of BRD4 in controlling the SASP and its ability to mediate senescent cell clearance. It also highlights the fact that not all SASP is detrimental, but it is unclear what SASP components are beneficial and which ones are detrimental, and to what extent restoring the beneficial ones to tumor cells could prove useful in re-triggering the immune surveillance program.

## SENESCENCE IN AGING AND CANCER

Cellular senescence has long been acknowledged as a potent anti-cancer mechanism that establishes a stable and irreversible cell cycle arrest of precancerous cells [14]. While these intrinsic tumor suppressive properties are clearly advantageous, recent evidence suggests that senescent cells are not genomically stable, and the mere presence of senescent cells and their SASP can act extrinsically to promote neoplastic transformation of premalignant cells [15, 16]. This double-edged sword of tumor protection and promotion is beginning to be understood and it has become imperative to determine how to keep the benefits of senescence intact while avoiding the deleterious consequences. A limitation to understanding the mechanistic underpinnings of senescence and the SASP is that many studies have been performed in tissue culture where a large number of relatively uniform senescent cells can be used for analyses. Identification and characterization of senescent cells in tissues is complicated by the fact that a single, distinguishing, unique factor of senescence has not been established. Identifying novel biomarkers and means of isolating and characterizing this rare cell type from aged and diseased tissues will greatly enhance our understanding of how these cells promote dysfunction.

**John Sedivy** (Brown University; Providence, USA) discussed the epigenetic alterations that occur in senescent cells. He highlighted his recent publication that used replicatively senescent human diploid fibroblasts (HDFs) for whole-genome chromosome conformation (Hi-C) assessments in combination with fluorescence *in situ* hybridization (FISH) painting to establish the three-dimensional chromatin architectural alterations and to map the chromosomal territorial shifts that occur in senescence [17]. They found that while there is generalized compaction and shrinkage of chromatin, senescent cells tend to gain close range interactions and lose long-range interactions. This compaction pattern coincides with a reduction in total volume occupied by chromosome arms. Interestingly, the highly repetitive (and retrotransposable elements-rich) regions of centromeres and peri-centromeres become enlarged and distended in senescent cells. Using formaldehyde assisted isolation of regulatory elements (FAIRE), the Sedivy team shows that chromatin profiles of senescent cells exhibit a generalized loss in signal at normally open, nucleosome-poor enhancers and promoters of active genes, while gaining signal in gene-poor heterochromatic regions [18]. The consequence of this is that transcription and translation of highly repetitive satellites and retrotransposons increases in senescent cells, which is in accordance with an increase in centromeric and peri-centromeric volume in senescent cells. Further tests also indicate that transposition in senescent cells requires three distinct alterations, including loss of RB, loss of TREX1 endonuclease and a gain of FOXA1, which collectively drive Line-1 transcription, transposition and associated DNA damage and senescence. Interestingly, if these factors are restored to normal in senescent cells, transposition is stopped. Dr. Sedivy then redirected the discussion to how Polycomb Group (PcG) chromatin regulators might influence epigenetic alterations in senescent cells. Based on recent developments in his lab, he proposed a “WNT-MYC-EZH2 axis” model, where loss of members from this pathway promotes senescence. Enhancer of Zeste Homolog 2 (EZH2), a PcG histone methyltransferase of H3K27, is reduced in senescent cells, which leads to an induction of the *INK4A-ARF* locus [19]. Dr. Sedivy's group has found that the downregulation of EZH2 promotes cellular senescence through a bimodal and complementary set of mechanisms. On the one hand, loss of EZH2 downstream of reduced WNT-MYC axis signaling leads to H3K27me3-independent activation of a rapid and robust ATM/p53/p21^Cip1^-dependent DNA damage response and S-phase checkpoint. This is likely due to DNA damage that incurs from DNA replication fork collapse in the absence of functional Polycomb complexes. On the other hand, loss of EZH2 methyltransferase activity will cause a gradual loss of H3K27me3, which in turn results in the de-repression and induction of p16^Ink4a^ and SAβG activation, along with mTOR/NFκB-mediated production of the SASP. He closed with a “S-phase vulnerability” hypothesis requirement for entry into senescence, where mitotically competent cells experiencing prolonged/unresolvable stresses accumulate DNA damage and enter a permanent state of cell cycle arrest.

**Sheila Stewart** (Washington University School of Medicine; St. Louis, USA) began by acknowledging that cancer predisposition is inherently linked to aging, however the mechanisms underlying this link are relatively unknown. Intrinsic changes in mutational load and stochastic senescence of cells increases dramatically with age, suggesting that they may facilitate the development of late-life cancer and associated metastasis. Using the FASST mouse (Fibroblasts Accelerate Stromal-Supported Tumorigenesis) model with an inducible LSL-p27^Kip1^ cassette knocked into the ROSA26 locus, they are able to induce p27^Kip1^-driven senescence specifically in osteoblasts once these mice are crossed to a pro-alpha 2(I) collagen promoter-driven Cre-ER transgenic. Intracardial injection of isogenic NT2.5 murine breast cancer cells into this FASST mouse results in increased osteoclastogenesis and bone metastases that correlates with enrichment for p27^Kip1^-induced osteoblast senescence (i.e., SAβG^+^) and SASP expression (i.e., IL6^+^, among others) [20]. Conversely, neutralization of IL6 as a key SASP factor is sufficient to limit the effects of osteoblast senescence and SASP in attracting breast cancer cells to the bone and consequently reducing the tumor burden [20]. Additionally, Dr. Stewart presented data on how senescent stromal cells may impact primary tumor growth. Here the focus was on skin and senescent dermal fibroblasts [21]. While it is known that senescent fibroblasts accumulate with age in mouse and human skin alike, data from the Stewart lab points to the concomitant clustering and co-localization of CD45^+^ immune cells near the FASST-induced senescent fibroblasts and in the aged murine and human skin. Further analysis revealed that cells known to suppress the adaptive immune system – such as immature granulocytes and regulatory T cells – are increased in senescent/aging stroma. Interestingly, treatment of bone marrow cells with either FASST mouse-derived senescent or non-senescent fibroblast CM resulted in a significant expansion of immature granulocytes in the group cultured with senescent fibroblast CM. Furthermore, senescent fibroblast and CM-educated granulocytes are able to suppress CD8^+^ cells in co-culture assays, unlike the non-senescence CM-educated cells. These effects are recapitulated *in vivo*, where tumor growth of isogenic murine SCC lines (PDSC5 and MK16-Ras) only occurs when they are co-injected sub-cutaneously onto FVB mice with senesced murine dermal fibroblasts – yet are readily blocked upon depletion of IL6 and/or immature granulocytes via αIL6 (or fibroblast-specific shRNA) and/or αLy6G neutralizing antibody treatment. This protective effect is reversed if αCD8 antibodies are administered, and establishes a link between stromal senescence, SASP-mediated immune suppression and cancer [21]. Together, these results suggest that changes in the distal or primary site microenvironment due to components secreted from senescent cells promote a permissive and metastatic niche that facilitates tumor growth and cell engraftment.

In the final talk of this session, **Jan Vijg** (Albert Einstein College of Medicine; New York, USA) discussed the challenges faced in the field to accurately measure genome instability during aging and in senescence at the single cell level. In collaboration with Cristina Montagna's group, Dr. Vijg's team developed a combination of assays to accurately detect single nucleotide variations (SNV) and genome structural variations (SV) in individual cells – events that may otherwise be diluted/subtracted out when analyzed in aggregate approaches. Published FISH and fluorescence-activated cell sorting (FACS) analysis of cerebral cortex-derived cells show that aneuploidy is not uncommon, increases with age (as demonstrated for mouse chromosomes 7, 18 and Y) and is concentrated in replication competent glial cells as opposed to non-dividing neurons [22]. Follow up studies using the novel SNV/SV technologies on a variety of human (IMR90 and BJ fibroblasts, B- and T-cells) and murine (mouse embryonic fibroblasts) cell types support the notion that, at the single cell level, the mutational load (involving chromosome gains/losses, point mutations, and genome structural variations) increases with senescence and aging. This also confirms what has been observe and documented by others [23], but at a much more informative scale that enables the linkage of specific mutation events on a per cell basis to senescence and aging as biological endpoints. Together these results suggest that careful analysis of individual senescent cells for genomic instability are technically possible and may illuminate how senescence and genome instability are linked.

## DETRIMENTAL EFFECTS OF THERAPY-INDUCED SENESCENCE

Chemotherapeutic strategies designed to kill a high percentage of cancer cells are increasingly being shown to induce cellular senescence *in vivo* in a variety of cells, both cancerous and non-cancerous [4]. Over time, these therapy-induced senescent cells evade clearance by the immune system and accumulate in the body, where they have the potential of producing a SASP [24]. Ironically, this SASP-enriched condition may in fact create a microenvironment within tissues that favors cancer growth [4, 24]. Equally as significant, there is growing appreciation for how genetic polymorphisms in differing populations may dictate cellular effects/outcomes to standard anti-cancer therapeutics.

**Maureen Murphy** (The Wistar Institute; Philadelphia, USA) discussed the functional significance of a single nucleotide polymorphism of the tumor suppressor p53 that is found in a subset of African Americans [25]. Typically, codon 47 of p53 encodes for the amino acid proline (P), but they found a polymorphism that alters this proline (P47) to a serine (S47). The S47 variant impairs the phosphorylation status of S46 that is mediated by proline-dependent kinases, including p38MAPK, HIPK2 and DYRK [25]. To understand the functional significance of the S47 variant *in vivo*, they utilized the humanized p53 knock-in (Hupki) p53 allele to develop a S47-specific knock-in mouse. Wild-type (WT) human or mouse p53 triggers potent cell death in response to genotoxic agents including etoposide, doxorubicin, and γ-irradiation (IR). Interestingly, S47 cells (exemplified by Hupki mouse embryonic fibroblasts and human-derived and EBV-immortalized polymorphic lymphoblast cell lines) show only a modest reduction in cell death upon exposure to these stresses. Surprisingly, there are no marked differences in growth arrest or transactivation of most p53 target genes between p53 wild-type and S47 cells [25]. However, the S47 variant appears to have increased reactive oxygen species (ROS) and senescence, which is accompanied by OXPHOS → Glycolytic metabolic shift (i.e., Warburg Effect) and extreme resistance to cisplatin-induced cell death. Mechanistically, cisplatin induces cell death in part by ferroptosis, an iron mediated non-apoptotic cell death that is driven by p53. Resistance of the S47 variant to ferroptosis was attributed to its impaired ability to transactivate a subset of p53 target genes involved in metabolism and ferroptosis, GLS2 (glutaminase 2), and SCO2 (cytochrome c oxidase assembly protein). In line with the S47 variant's defect in the tumor suppressive function of ferroptosis, both homozygous and heterozygous S47 mice are more prone to various types of cancer (and metastasis), including pancreatic cancer, colorectal cancer, hepatocellular carcinoma and the extremely rare histiocytic sarcoma [25]. Taken together, this work identified a functional role for the S47 variant *in vivo*, which may, in part, underlie the increased susceptibility of African Americans to carcinogenesis and contribute to its disparities-linked aggressiveness and resistance to therapy.

**Igor Roninson** (University of South Carolina; Columbia, USA) discussed the importance of modulating chemotherapy-induced senescence and SASP-related paracrine effects that may interfere with treatment efficacy. He argues that the secretory phenotype associated with DNA damaging agents like doxorubicin results in part from CDK8-mediated transcriptional reprogramming. CDK8 (and its paralog CDK19) can be stimulated by p21^Cip1^ or other CDK-binding proteins. While p21^Cip1^ binds to and blocks several cell cycle progression CDKs, it also forms a complex with CDK8 and promotes the induction of tumor-promoting cytokines. DNA damage induced by different drugs is accompanied by CDK8/19 transcriptional activation of genes involved in the SASP, including cytokines, angiogenic factors and mitogens, which ultimately protect tumor cells from chemotherapeutic death [26]. High throughput screening for candidate compounds downstream of DNA damage-induced p21^Cip1^, followed by chemical lead optimization, identified Senexin A (and the more potent second generation Senexin B) that selectively binds to and blocks the ATP pocket of CDK8/19. *In vitro*, Senexin inhibits the secretion of anti-apoptotic factors by doxorubicin-treated or γ-irradiated cells, and also blocks the induction of cytokines downstream of the TNFα-NFκB axis. Equally as significant, Senexin interferes with Wnt3a/β-catenin and TGFβ axes-mediated transcription of invasion/migration genes. The *in vivo* effects of Senexin are as promising, where treatment of xenograft mouse models inhibits the growth of breast and prostate cancers, and markedly suppress colon cancers metastasis. The beneficial effects of Senexin are further enhanced by preventing CDK8-mediated activation of STAT1 and its downstream suppressive effects on NK cell immune surveillance functions. These findings support the development of drugs such as CDK8/19 inhibitors, which attenuate the senescence-associated tumor-promoting properties of chemotherapy.

**Ashani Weeraratna** (The Wistar Institute; Philadelphia, USA) also argued that senescence is not necessarily a good therapeutic endpoint for a couple of reasons. First, universal markers of senescence have not yet been identified. This makes it difficult to discern true senescence from pseudo-senescence that may arise from and respond differently to cancer therapy and contribute to tumor heterogeneity. Second, senescent cells may (and often do) secrete factors that promote invasion of surrounding cells and contribute to a pro-metastatic microenvironment that exacerbates with age. In melanoma, cells appear senescent after exposure to stressors such as irradiation (IR) or drug therapy [27]. These pseudo-senescent cells are growth arrested, express classical senescent markers such as SAβG, PML bodies, and heterochromatin foci, yet, they are termed pseudo-senescent because they are resistant to therapy and acquire a highly invasive and pro-metastatic phenotype. Mechanistically, non-canonical Wnt5a appears to drive pseudo-senescence. Compared to low Wnt5a expressing melanoma cells, high Wnt5a expressing cells appeared to senesce in response to a variety of therapeutically-relevant stressors (IR, B-Raf inhibitors) and subsequently acquired both invasive and metastatic potential. Conversely, knock-down of Wnt5a in high expressors blocks senescence and metastasis, suggesting that Wnt5a is a critical regulator of pseudo-senescence [27]. Dr. Weeraratna further argued that senescence, within the context of aging and the aged microenvironment, also contributes to more aggressive tumors. *In vitro*, melanoma cells embedded in skin equivalents reconstituted with aged (55-65 year-olds) patient-derived dermal fibroblasts are significantly more invasive than organotypic cultures embedded with young (25-35 year-olds) dermal fibroblasts. Similarly, the aged microenvironment of C57BL/6 mice promotes melanoma progression to metastasis and resistance to chemotherapy. Tail vein injection of YUMM1.7 (Braf^V600E^/Cdkn2a^−/−^/Pten^−/−^) mouse melanoma cells into young (8 weeks) or aged (52 weeks) mice, resulted in smaller but substantially more aggressive and metastatic tumors in the aged mice, which is likely mediated by secreted proteins in the aged microenvironment [28]. Secretome analysis identified Secreted Frizzled Related Protein (SFRP2) – an inhibitor of canonical Wnt-β-Catenin signaling – as a pro-invasive and angiogenic factor in melanoma. Moreover, SFRP2 protein levels increase with age; and induction of SFRP2 (and loss of β-Catenin) in aged skin further exacerbated the tumor-permissive and therapy-resistant qualities of the aged microenvironment by enhancing DNA damage and oxidative stress through the inhibition of the redox effector APE1 and microphthalmia associated transcription factor [28]. Together, these findings suggest a role for senescence in creating an aggressive tumor microenvironment in aged skin, warranting the development of therapeutics aimed at eliminating the detrimental effects of senescent cells.

**Lynne Elmore** (Virginia Commonwealth University; Richmond, USA) emphasized the clinical importance of better understanding the relationship between therapy-induced senescence and cancer recurrence; and whether therapy-induced senescence in cancer cells is reversible. This is in spite of the fact that lineage-tracing technologies have been lacking in the field, which makes single cell fate determination a challenge. To this end, the David Gewirtz laboratory (in collaboration with Dr. Elmore's team) then developed a single cell approach utilizing flow cytometry to enrich for and monitor individual senescent cancer cells over time using C12FDG, a fluorescent method to detect SAβG. After exposure of cancer cell lines to the chemotherapeutic agent etoposide, cells that were concluded to be senescent (based on growth arrest and SAβG positivity status) were sorted and analyzed for re-entry into a proliferative state. Over a period of days (between 10-20 days post-treatment), individual senescent cancer cells lost SAβG positivity and resumed proliferation, suggesting that these cells recovered from etoposide-induced senescence – a condition that may ultimately facilitate cancer recurrence. Verifying whether and how senescence in cancer cells is reversible, both in cell culture and *in vivo*, will help design strategies to eliminate cancer relapse in patients who have previously been treated with senescence inducing chemotherapeutic drugs. Dr. Elmore concluded by touching upon her interest in identifying the mechanistic role of different SASP factors and microRNAs in regulating senescence reversal in cancer cells, as these may be novel therapeutic targets to prevent cancer recurrence.

## FROM CELLULAR SENESCENCE TO CANCER *IN VIVO*

Senescence is an effective barrier to cancer progression as it limits the proliferation potential of damaged cells. What is less understood is why a damaged cell enters senescence rather than apoptosis. From a cancer perspective, apoptosis would seem to be more advantageous, because it prevents tumorigenesis without inflammation – yet senescence is a common consequence of oncogenic insults. Understanding why senescence occurs could lead to deeper mechanistic insights into its role in cancer. The inherent complexities associated with cell autonomous and non-autonomous effects of senescence and the SASP has made it essential to develop more sophisticated *in vivo* systems in order to comprehensively study its multifaceted biological significance.

**Gregory David** (New York University School of Medicine; New York, USA) began this session by emphasizing that the contributions of senescent cells and the SASP-related inflammatory response to cancer progression is context-dependent. Dr. David's research team focuses on Sin3B, a component of the Sin3-HDAC co-repressor complex that they found is required for stress-related senescence induced by either gain of oncogenic KRAS or loss of PTEN in prostate and pancreatic cancer models, respectively [29]. On the one hand, senescent cells that emerge due to oncogenic stress in prostate cancer platforms appear to function in a tumor-suppressive manner. Compound prostate-specific PTEN^−/−^Sin3B^−/−^ mice, for instance, are blocked from developing and accumulating senescent cells and have increased tumor-associated morbidity and mortality relative to PTEN^−/−^ mice. On the other hand – and in stark contrast – pancreas-specific (and senescence deficient) KRAS^G12D^Sin3B^−/−^ mice have appreciably delayed conversions of pancreatic intraepithelial neoplasia to ductal carcinoma (PDAC) and PDAC-associated death [30]. Significantly, the SASP is also reduced, along with a reduction in immune infiltrates and inflammation. Specifically, deletion of Sin3B results in downregulation of key pro-inflammatory cytokines, including IL1α and its downstream targets IL6 and IL8. Moreover, depletion of IL1α in KRAS^G12D^ mice phenocopies the tumor-protective effects observed with the loss of Sin3B. This demonstrates that the Sin3B-SASP-IL1α axis is critical for pancreatic cancer progression. Interestingly, knockdown of IL1R leads to downregulation of the SASP, yet IL1α depletion does not affect the induction of senescence per se (as determined by multiple markers, including heterochromatin foci formation). Dr. David concluded by indicating that delineating the fate and context-dependent effects of senescent cells during cancer progression will require lineage-tracing platforms currently unavailable to the community at large.

**Vera Gorbunova** (University of Rochester; Rochester, USA) has recently embarked on studying the effects of senescence in the long-lived and tumor-resistant naked mole rat (NMR). Similar to mice (but unlike humans), NMR somatic cells express telomerase, albeit at lower levels, and are not amenable to telomere-dependent replicative senescence. Nevertheless, they do undergo developmental senescence and respond mildly to UV- and IR-induced senescence. It remains unclear whether this inherent resistance to senescence plays a role in extending the lifespan of NMRs – or whether it helps protect against senescence-driven tumors, as others have argued. Significant findings by the Gorbunova lab may help clarify the biological significance of NMR's inherent resistance to senescence. First, Dr. Gorbunova found that, unlike most mammals, the NMR INK4 locus codes for pALT^Ink4a/b^, a potent cyclin-dependent kinase inhibitor (CDKi) [31]. pALT^Ink4a/b^ is an alternatively spliced hybrid of p15^Ink4b^-p16^Ink4a^ comprised of exon 1 of p15^Ink4b^ and exons 2 and 3 of p16^Ink4a^. Like its counterparts within the INK4 locus (p15^Ink4b^, p16^Ink4a^, and p19^Arf^), pALT^Ink4a/b^ is induced by contact inhibition and/or genotoxic stresses. Second, NMRs synthesize high molecular mass hyaluronan (HA) which, unlike the five times smaller low molecular mass HA expressed by mouse and humans, makes dermal fibroblasts hypersensitive to contact inhibition. Mechanistically, high molecular mass HA signals the expression of pALT^Ink4a/b^ through CD44. Production of high molecular mass HA by NMRs confers cancer resistance and restricts proliferation [32]. NMR cells are normally resistant to SV40 LT + HRas^V12^ transformation, but shRNA knockdown of HA Synthase (HAS2) in NMR dermal fibroblasts co-transfected with LT and Ras are prone to growth in soft agar and tumor development in xenograft mouse models. NMR-HAS2 transgenic mice have been developed recently and will be instrumental in determining how HA may impact aging, senescence, cancer and lifespan.

**Norman Sharpless** (University of North Carolina School of Medicine; Chapel Hill, USA) discussed ways his team has used p16^Ink4a^ as a biomarker to measure the “molecular/physiologic age” of animal models and people, which is a different measure than chronological age. In humans, the *INK4/ARF* locus is strongly associated with a variety of age-related diseases, including myocardial infarction, stroke, diabetes, glaucoma, aneurysms, and cancers. Unlike other CDK inhibitor proteins such as p21^Cip1^ and p27^Kip1^, the expression of p16^Ink4a^ and p19^Arf^ increases with age in both mice and people. Conversely, age-prolonging caloric restriction limits increases in p16^Ink4a^ levels. Dr. Sharpless argues that the expression of p16^Ink4a^ strongly correlates with the overall burden of senescent cells and serves as a biomarker of physiologic age. For instance, serial measurements of p16^Ink4a^ levels in a p16^Ink4a^-luciferase knock-in mouse model developed in his lab shows that p16^Ink4a^ levels increase exponentially with age. Parallel measurements of p16^Ink4a^ in humans with a recently developed peripheral blood-based CD3^+^ T-cell assay show that on average, p16^Ink4a^ mRNA levels increased approximately 1.5 fold per decade of life – an increase that begins well in advance of the manifestation of “aging phenotypes”. This ultimately results in a 20-fold increase in senescence positivity over an eight-decade adult lifespan. To what extent the accumulation of p16^Ink4a^-positive senescent cells is a result of age-related decline in immune surveillance and/or of SASP-related bystander effects remains unclear, but mathematical models of the observed exponential growth fits well with senescent cells promoting the accumulation of more senescent cells. Various extrinsic factors were also tested for their effect on p16^Ink4a^ induction, including cigarette smoke, exercise and chemotherapeutic agents. The number of pack-years of smoking correlates with increases in p16^Ink4a^, while the length of exercise sessions inversely correlates with p16 status. Within the clinical realm, adjuvant chemotherapy of breast cancer patients with adriamycin and cytoxan is shown to accelerate p16^In4a^-based molecular aging. In turn, there is also preliminary evidence that the p16^Ink4a^ pre-treatment status in patients can be predictive of degree of chemotherapy toxicity – i.e., the higher the baseline, the higher the toxicity. Dr. Sharpless believes that utilization of a diagnostic test to measure the amount of p16^Ink4a^ in peripheral blood lymphocytes, which is commercially available, may provide insight into the “molecular age” of patients to provide guidance for therapeutic decisions.

## TUMOR IMMUNOLOGY AND SENESCENCE

The last session focused on how immune cell function may interact with senescent cells to contribute to tumorigenesis. This session further highlighted the importance of having robust *in vivo* platforms in which to study the interplay between senescence, SASP and immune surveillance programs within the context of the aging and/or tumor microenvironment. Many of these mechanisms are beginning to emerge, and a better understanding of how senescent cells are detected and selectively eliminated before they disrupt normal tissue functions will have wide-ranging translational ramifications.

**Lars Zender** (University Hospital Tübigen; Tübigen, Germany) discussed the complex interplay between oncogene-induced senescence and immune surveillance in liver tumorigenesis and progression. Published work from his lab shows that *in vivo* liver tumor regression mediated by WT-p53 re-activation in HRas^G12V^ p53^−/−^ hepatocytes involves the induction of senescence, SASP and immune cell recruitment into the tumor microenvironment – not apoptosis [33]. Consistent with these findings, senescence-induced immune surveillance is also relevant to the elimination of pre-malignant NRas^G12V^ lesions [34]. Mechanistically, rampant senescence and resulting SASP stimulate the clearance of senescent cells by attracting CD4^+^ T-cells and CCR2^+^ inflammatory monocytes. Likewise, NK cells also contribute to this coordinated clearance, as loss of NK cells accelerates tumorigenesis [33, 34]. Furthermore, antibody-mediated depletion of immune cells compromises the SASP-activated surveillance, and enable hepatocellular carcinomas (HCC) to develop and progress unhindered. More recently, secretome analysis of the SASP revealed that senescent pre-malignant hepatocytes express very high levels of MCP1 (CCL2). Mice deficient for the CCL2 receptor (CCR2, expressed in inflammatory monocytes, hematopoietic stem cells and subsets of NK cells) have impaired infiltration and maturation of bone marrow-derived immature myeloid cells (iMC) into macrophages in the senescence-rich liver. This in turn impairs the coordinated clearance of pre-cancerous senescent hepatocytes by the macrophages and CD4^+^ T-cells. The accumulation of iMC in the liver has the added negative effect of blocking NK cell function – which further compromises the senescence surveillance program. Without appropriate immune clearance, NRas^G12V^ cells may stochastically bypass senescence, become fully tumorigenic, and further fuel iMC maturation/functional blockade via poorly understood tumor-secreted factors. In line with this observation, peritumoral senescence in both mice and humans track with poor HCC prognosis and survival, and support the notion that retention/accumulation of senescent cells exacerbate the tumor phenotype and is deleterious long-term [35]. Basic mechanistic studies as the ones uncovered in the Zender lab may help better understand the ramifications of targeted therapies for a wide range of senescence-associated diseases. CCR2 inhibitors, for instance, have recently entered clinical trials for patients with diabetic nephropathy and Type 2 diabetes. Sadly, liver damage (often observed as a precursor to cancer) has been reported for these patients, and the extent to which it is linked to blocked senescence surveillance remains to be seen.

**Daohong Zhou** (University of Arkansas; Little Rock, USA) discussed how cancer therapies, including chemotherapy and ionizing radiation (IR), often induce cellular senescence. The induction of senescence in response to these signals is beneficial in the short term, as it limits the proliferation of damaged cells. However, these cells are becoming increasingly recognized as deleterious in the long term, as a growing body of evidence suggests that the accumulation of senescent cells contributes to tissue damage and facilitates tumor recurrence and metastases. Dr. Zhou has been focused on discovering pharmacological interventions that can target senescent cells, so called “senolytic” agents, to selectively eliminate them to improve health and the efficacy of cancer treatments. One such drug, ABT-263 (Navitoclax), a specific Bcl-2 and Bcl-xl inhibitor, is broadly senolytic in multiple cell types induced to senesce in several ways [36]. Cell death upon ABT-263 treatment is dependent on activation of caspases. Administration of ABT-263 to both aged and sublethally irradiated mice depleted senescent hematopoietic stem cells (HSCs) and satellite (skeletal muscle stem) cells (MuSCs), which resulted in mitigation of myeloid skewing bias from HSCs and improved clonogenicity of MuSCs. Dr. Zhou pointed out that ABT-263 is not a “fountain of youth”, as this drug has significant toxicity and lacks the ability to kill all senescent cells. However, preliminary studies suggested that survival may be extended with ABT-263 treatment. It will be interesting to see how pharmacological interventions targeting senescent cells, including ABT-263 and other senolytic drugs, impact life and healthspan.

## WORKSHOP SUMMARY

At the conclusion of the workshop, a round table discussion led by Judy Campisi (Buck Institute) and Jan van Deursen (Mayo Clinic) focused on defining the major challenges and opportunities in this emerging research area. It was noted how far the field has come in the past two decades, during which senescence was viewed as a tissue culture “phenomenon” that had little role in physiology. The discussion focused on resources, tools and technologies, knowledge gaps, and research community efforts that will be required to accelerate discoveries. Key points are summarized below.

### How to properly define senescence

- The term senescence is used to define a cellular phenotype that has arisen through various means, including developmental cues, tissue damage and the stresses of aging. Often the underlying reason for a cell becoming senescent is unknown or unclear, yet the outcomes are grouped into one category. In publications, these caveats are not stated, although they may impact the conclusions.
- Should the field establish criteria for concluding that a cell is senescent? Or is it favorable to have a malleable definition of senescence? There was no agreement about whether there should be a minimal, rigid criterion or whether a more fluid definition is preferred.

### Overcoming knowledge gaps

- Senescence in culture can be a highly dynamic process as exemplified in what many refer to as “deep-senescence”. Is this process also dynamic *in vivo* and what methods are needed to test the idea that senescence evolves over time in an organism? In addition, there are no experimental designs that allows one to conclusively demonstrate that senescence bypassing promotes intrinsic transformation of permanently arrested cells *in vivo*. Efforts should be made to define what “bypassing senescence” actually entails from a molecular standpoint. To state the problem more simply, is senescence actually reversible and is it possible for all forms of senescence?
- The contribution of senescent cells to tissue and organ phenotypes *in vivo* is beginning to be elucidated, but it is unclear whether and how metabolic alterations influence senescent phenotypes and the SASP. We also do not know how many senescent cells accumulate with age in each tissue, nor how they promote cancer.
- Furthermore, defining the cell autonomous effects of the senescence response versus the impact on the tissue microenvironment needs careful examination.
- Senescence can be beneficial or detrimental, depending on the context. Establishing how senescent cells contribute to degenerative diseases or cancer may be necessary to determine whether removal or ablation strategies should be utilized. In certain cancers, senescent cells may actively promote tumorigenesis and resistance to therapy. These would be the types of tumors to first test whether senolytic interventions are beneficial.

### Tool and resource development

- Clearly the field needs more advanced tools, although it is unclear what this tool kit would look like. One important tool that needs development is the ability to follow and lineage trace senescent cells over time *in vivo*, which would help our understanding of their etiology and whether (and to what extent) the senescent state is reversible. In addition, improved animal models are needed to better understand the contribution of senescent cells to cancer, including the development of mouse models that allow for the isolation of senescent cells from tissues.
- More sophisticated tissue culture models are also needed not only to determine how oncogenic senescence arises, but also to identify and characterize the corresponding SASP elements/signatures. A variety of cell types should be used to determine a “core” group of SASP factors that might be universal.
- The field should work to establish a common platform for defining the SASP and senescent states, both in culture and *in vivo*. This task may require single cell RNA analyses, which would be greatly facilitated by the development of robotics to perform these analyses. Using these tools, one would be able to build signatures of the SASP in different senescent states and cell types.

### Comparative studies

- Studies performed in culture and in lower organisms will inform our understanding of oncogenic senescence in humans. Uniting the basic and clinical sciences is a major challenge to this field, but clearly is required to translate the conclusions gained from model systems into therapeutic opportunities in patients. Efforts to support these interactions should be encouraged. It is possible that clinical applications can be developed despite uncertainties about how senescent cells drive disease.

### Team building

- Rapid progress in the field will require the efforts of collaborative teams, ranging from basic scientists to clinicians, with complementary skillsets and model systems. Addressing these critically important needs will facilitate scientifically rigorous experimentation to fully explore this emerging research area and elucidate the relationship between senescence and carcinogenesis.

### Attendees

Fatouma Alimirah (Buck Institute for Research on Aging; Novato, CA), Darren Baker (Mayo Clinic; Rochester, MN), Ana Banito (Memorial Sloan-Kettering Cancer Center; New York, NY), Judith Campisi (Buck Institute for Research on Aging; Novato, CA), Gregory David (New York School of Medicine; New York, NY), Lynne Elmore (Virginia Commonwealth University; Richmond, VA), Daniel Gallahan (National Cancer Institute, NIH; Bethesda, MD), Vera Gorbunova (University of Rochester; Rochester, NY), Jeffrey Hildesheim (National Cancer Institute, NIH; Bethesda, MD), Bill Keyes (Centre for Genomic Regulation; Barcelona, Spain), Maureen Murphy (The Wistar Institute; Philadelphia, PA), Igor Roninson (University of South Carolina College of Pharmacy; Columbia, SC), John Sedivy (Brown University; Providence, RI), Manuel Serrano (Spanish National Cancer Center (CNIO); Madrid, Spain), Norman Sharpless (University of North Carolina School of Medicine; Chapel Hill, NC), Dinah Singer (National Cancer Institute, NIH; Bethesda, MD), Barbara Spalholz (National Cancer Institute, NIH; Bethesda, MD), Sheila Stewart (Washington University School of Medicine; St. Louis, MO), Jan van Deursen (Mayo Clinic; Rochester, MN), Jan Vijg (Albert Einstein College of Medicine; Bronx, NY), Ashani Weeraratna (The Wistar Institute; Philadelphia, PA), Lars Zender (University Hospital Tübigen; Tübigen, Germany), Daohong Zhou (University of Arkansas; Little Rock, AR).
